# Ulnar-Sided Sclerosis of the Lunate Does Not Affect Outcomes in Patients Undergoing Volar Locking Plate Fixation for Distal Radius Fracture

**DOI:** 10.3390/jcm12186003

**Published:** 2023-09-16

**Authors:** Jong-Hun Baek, Jae-Hoon Lee, Ki-Hyeok Ku

**Affiliations:** 1 Department of Orthopedic Surgery, Kyung Hee University School of Medicine, Kyung Hee University Hospital, Seoul 02447, Republic of Korea; paeton81@naver.com; 2 Department of Orthopedic Surgery, Yeson Hospital, Bucheon 14555, Republic of Korea; ljhos69@naver.com; 3 Department of Orthopedic Surgery, Graduate School, Kyung Hee University School of Medicine, Kyung Hee University Hospital at Gangdong, Seoul 05278, Republic of Korea

**Keywords:** distal radius fracture, ulnar impaction syndrome, ulnar variance, volar locking plate fixation

## Abstract

Background and aim: Radial shortening after distal radius fracture causes ulnar impaction, and a mild reduction loss of radial height occurs even after volar locking plate fixation. This study aimed to determine whether preoperative ulnar-sided sclerosis affects clinical outcomes after volar locking plate fixation for distal radius fracture (DRF). Method: Among 369 patients who underwent volar locking plate fixation for DRF, 18 with preoperative ulnar-sided sclerosis of the lunate were included in Group A and compared to a 1:4 age-, sex- and fracture-pattern-matched cohort without sclerosis (72 patients, Group B). The visual analog scale (VAS), Disabilities of the Arm, Shoulder, and Hand (DASH) score, and grip strength were assessed as clinical outcomes. Ulnar variance (UV), radial inclination, radial length, and volar tilt at two weeks after surgery and the final follow-up were measured as radiographic outcomes. Results: The mean VAS and DASH scores and grip strength did not differ between the two groups. The mean UV at two weeks after surgery and the last follow-up was significantly higher in Group A. The mean changes in UV were +0.62 mm in Group A and +0.48 mm in Group B. There were no significant intergroup differences. Neither UV nor its changes showed any association with DASH and VAS scores. Conclusions: Preoperative ulnar-sided sclerosis of the lunate did not affect clinical outcomes after volar locking plate fixation, even if UV increased postoperatively.

## 1. Introduction

Distal radius fracture (DRF) is quite common, accounting for approximately 3% of all upper-extremity fractures [1]. The recently introduced volar locking plate fixation (VLPF) for DRF became a widely-used treatment option with a reliable fixation and low complication rates [2]. However, ulnar-sided wrist pain is reported at a rate of 15% even after VLPF, and the cause of it is of great interest [3]. In patients with DRF, subchondral sclerosis with or without cystic changes in the proximal ulnar portion of the lunate is often found on preoperative plain radiography. Although ulnar impaction syndrome (UIS) is usually diagnosed with specific physical examinations and radiographic evaluations, many studies have reported that the presence of subchondral sclerosis in the proximal ulnar portion of the lunate is among the most important radiographic findings of UIS [4,5,6,7]. 

UIS is a painful degenerative condition that results from the abutment of the distal end of the ulna against the ulnar carpus. This syndrome may occur in wrists with positive ulnar variance (UV), which can be caused by malunion after DRF, such as radial shortening and loss of volar tilting [8,9,10]. These types of malunion occur due to non-anatomic reduction and the gradual loss of reduction after DRF surgery and are often treated with ulnar shortening osteotomy or distal radius corrective osteotomy if symptoms persist [9,11]. In approximately 15% of patients undergoing ulnar shortening osteotomy, it is caused by ulnar impaction syndrome that occurred after VLPF of DRF [12,13]. In a cadaver study, wrists with ulnar positive variance are more vulnerable to radius malunion than wrists with an ulnar negative variant. Additionally, radial shortening of only a few millimeters changes the load distribution of the wrist [10]. Previous studies reported a positive change of 0.4–2.6 mm in UV compared to the immediate postoperative period after VLPF for DRF [14,15,16,17].

This study primarily aimed to determine whether preoperative ulnar-sided sclerosis of the lunate increases the risk of ulnar-sided wrist pain and affects clinical outcomes after VLPF for DRF. The secondary aim of this study is to determine whether a joint-leveling procedure would be required during surgery in patients with ulnar-sided sclerosis of the lunate in whom symptoms of UIS may manifest or worsen after VLPF for DRF. The authors hypothesized that patients with preoperative ulnar-sided sclerosis are vulnerable to a shortening of the radius of a few millimeters after VLPF. Furthermore, they hypothesized that postoperative symptoms of UIS, such as ulnar-sided wrist pain, may manifest or worsen according to positive changes in UV after VLPF for DRF.

## 2. Materials and Methods

### 2.1. Study Design and Patient Selection

**Figure 1:**
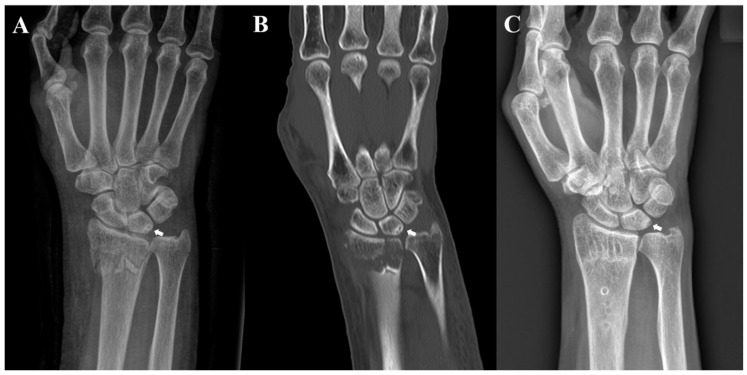
Radiographs of a 70-year-old woman. (**A**) Preoperative posteroanterior radiograph and (**B**) coronal computed tomography image revealing sclerosis with cystic changes on the ulnar side of the lunate; (**C**) posteroanterior radiograph acquired after the removal of internal fixation at 12 months after surgery reveals the remnant sclerotic lesion with cystic change of the lunate.

**Figure 2:**
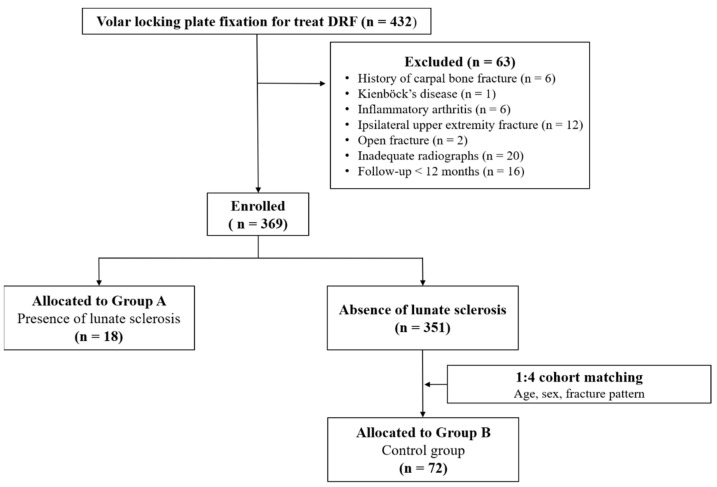
Flow diagram showing included and excluded patients. DRF—distal radius fracture.

Patients who underwent open reduction and internal fixation for DRF between July 2015 and December 2020 were retrospectively analyzed. During this period, 432 underwent VLPF for DRF. Among them, 63 were excluded due to meeting the following exclusion criteria: history of carpal bone fracture (*n* = 6), Kienböck’s disease (*n* = 1), or inflammatory arthritis (*n* = 6); presence of ipsilateral upper-extremity fractures including distal ulnar and shaft fractures, except ulnar styloid process fractures (*n* = 12), or presence of open fractures (*n* = 2); and inadequate radiographs for obtaining a true measurement (*n* = 20). Patients who were not monitored for more than 12 months were also excluded (*n* = 16). Finally, 369 patients were included in this study. Of them, 18 with subchondral sclerosis with or without cystic changes in the proximal ulnar portion of the lunate identified on preoperative plain radiographs and CT scans were included in Group A (Figure 1A–C). To uniformly match the age, sex, and fracture pattern with a standardized difference of <0.1, 72 patients from among the remaining 351 patients were included in Group B, which was the control group subjected to 1:4 cohort matching using nearest neighbor matching (propensity score matching) (Figure 2) (Table 1). This study was approved by our hospital’s Institutional Review Board (KHNMC 2021-02-012), which waived the requirement for informed consent. All patients’ data were made anonymous and kept confidential. All procedures were indicated and performed in compliance with our department’s standards and the Declaration of Helsinki.

### 2.2. Cinical and Radiographic Evaluations

Regular follow-up visits were performed at 2 weeks, 6 weeks, 12 weeks, 6 months, and 1 year after surgery. During follow-up, the patients were examined for pain by an operating surgeon (JHL). Pain levels were measured using a visual analog scale (VAS) ranging from 0 to 10 points. Among patients complaining of wrist pain, patients who had ulnar-sided wrist pain were asked whether the same pain had preceded a fracture and whether UIS had been diagnosed. Ulnar-sided wrist pain was defined as pain exacerbated by a forceful grip, pronation, and ulnar deviation. For patients who had ulnar-sided wrist pain, positive ulnocarpal stress test findings and tenderness were performed to determine whether the pain was caused by UIS at 6 and 12 months after surgery. The patients were asked to complete the Disability of the Arm, Shoulder, and Hand (DASH) questionnaire to assess patient-reported outcomes at 6 and 12 months after surgery. Grip strength was measured in both hands using a Jamar hydraulic hand dynamometer and calculated as a percentage of the unaffected side at 6 and 12 months after surgery. The right hand was assumed to be 15% stronger than the left hand in right-handed patients.

Fractures were classified according to the AO Foundation and Orthopaedic Trauma Association classification system [18]. The radiographic evaluations included posteroanterior and lateral views of the involved wrist for each patient. For the posteroanterior X-ray views, the shoulder was abducted 90° with the elbow flexed 90° and the wrist positioned in a neutral manner. In each group, UV, radial inclination (RI), radial length (RL), and volar tilt (VT) were measured 2 weeks and 12 months after surgery. On posteroanterior radiographs, UV was measured using two lines perpendicular to the axis parallel to the radial shaft, one at the distal end of the ulnar dome and the other at the lunate articular surface of the distal radius [19]. RI was measured as the angle between the line that was perpendicular to the radial shaft and the tangent to the distal radius of the articular surface. RL was measured as two lines perpendicular to the radial shaft, one drawn from the most distal part of the ulnar dome and the other from the radial styloid process. Finally, VT was measured on lateral radiographs. The angle was measured between the line connecting the volar and dorsal rims of the lunate articular surface of the distal radius and the line perpendicular to the long axis of the radius. Radiographic measurements were performed using a picture archiving and communication system (PiViewSTAR, Infinitt, Seoul, Republic of Korea). Radiographic parameters were measured independently by two experienced orthopedic surgeons (KHK and JHB). The intra- and interobserver reliabilities of all measurements were assessed using intraclass correlation coefficients. In this study, the intraclass correlation coefficient values of all measurements were greater than 0.8 for both intra- and interobserver reliabilities.

### 2.3. Statistical Analyses

Statistically significant intergroup differences were determined using independent t-tests after the Kolmogorov–Smirnov and Shapiro–Wilk tests, as appropriate, for normally distributed continuous variables. The last non-normally distributed UV and DASH scores were analyzed using the non-parametric Mann–Whitney U test. Fisher’s exact test was used to analyze categorical variables. The association between the UV and DASH scores was confirmed for each group. Spearman’s correlation was used to analyze the UV scores at the last follow-up, which were non-normally distributed. Immediate postoperative changes in UV that satisfied normality were analyzed using Pearson’s correlation coefficient. The relationship between the UV and VAS scores was assessed using Spearman’s correlation coefficient. A posteriori power analyses of the study and effect size were performed using the postoperative DASH scores of the two groups. The effect sizes (Cohen’s d) were calculated for clinical and radiologic outcomes. Statistical analyses were performed using the R software package (R version 3.5.1) and SPSS software (version 25.0; SPSS Inc., Chicago, IL, USA).

## 3. Results

The number of patients with ulnar-sided wrist pain was two (11.1%) in Group A and three (4.1%) in Group B. All patients were diagnosed with UIS before the fracture. None of the patients had newly diagnosed UIS or an exacerbation of ulnar-sided wrist pain. The mean VAS and DASH scores did not differ between the two groups at 6 and 12 months after surgery. The mean grip strength in Group A was slightly lower than that in Group B at 6 and 12 months after surgery. However, there were no significant intergroup differences (Table 2).

The mean UV at 2 weeks after surgery and at the last follow-up was higher in Group A than in Group B (Table 3). However, the mean changes in UV from 2 weeks after surgery to the last follow-up did not differ between the two groups. The mean RI, RL, and VT values did not differ between the two groups (Table 3). At the time of injury, posteroanterior radiographs of the contralateral normal wrists were available for 13 patients in Group A and 52 patients in Group B, while the mean UV of the normal wrist was 1.84 mm in Group A and 0.51 mm in Group B, showing a significant difference (*p* < 0.05).

The mean UV at 2 weeks after surgery and at the last follow-up and the change in UV showed no significant association with the DASH score (*p* > 0.05, >0.05, and >0.05, respectively). In addition, the UV and VAS scores showed no significant association (*p* > 0.05, >0.05, and >0.05, respectively).

## 4. Discussion

This study aimed to determine whether preoperative subchondral sclerosis with or without cystic changes in the proximal ulnar portion of the lunate is associated with an increased risk of ulnar-sided wrist pain or affects the clinical outcomes after VLPF for DRF. In our study, patients with ulnar-sided sclerosis of the lunate had a significantly larger UV value than those without. However, we could not confirm the clinical effects of preoperative ulnar-sided sclerosis of the lunate after VLPF for DRF and even increased postoperative UV values. Moreover, there were no correlations between UV and clinical outcomes, including VAS and DASH scores. Taken together, although ulnar-side sclerosis of the lunate occurred frequently in patients with positive UV findings, it did not manifest or worsen symptoms of UIS, even if UV increased slightly after VLPF for DRF.

A positive UV is a known risk factor for UIS because it increases the load on the ulnocarpal joint [20,21,22]. Palmar et al. reported that an increase of 2.5 mm UV increased the axial load in the ulnocarpal joint to 42%, while a decrease of 2.5 mm UV decreased the axial load to 4.3% [23]. RL shortening and VT loss after DRF can result in a positive UV [24]. Cheng et al. [16] reported a mean loss of UV of 1.3 mm after VLPF for extra-articular DRF. Arora et al. [14,15] reported a mean loss of UV and VT of 0.4–1.2 mm and 1.9–3.4°, respectively, after VLPF for DRF. Therefore, UIS can be caused by malunion after DRF, which can occur because of non-anatomical reduction or gradual loss of reduction after surgery. In this study, the mean changes in UV were 0.6 mm in Group A and 0.5 mm in Group B. These results were similar to those reported by Arora et al. and Cheng et al. Although the UV was significantly larger in Group A than in Group B, the clinical outcomes did not differ between the two groups. The VAS scores were 0.72 for Group A and 0.59 for Group B, which indicates very low pain scores in the two groups. Cheng et al. reported that the bigger change in radial height loss after VLPF showed a poorer clinical outcome in extra-articular-type distal radius fractures [16]. In the present study, the absolute length of UV is greater in group A than in group B. However, the postoperative UV changes between the two groups are similar. The similar postoperative UV changes between the two groups are a possible cause of these similar clinical outcomes between the two groups. UIS can also occur in cases of a neutral or even negative UV [25,26]. These findings indicate that an absolute value of positive UV was not a major cause of worsening UIS symptoms. Stefan et al. analyzed the functional and radiologic outcomes of 524 patients who underwent VLPF for DRF. They compared acceptable (<2 mm) and unacceptable (>2 mm) UV groups and found no significant differences [17].

Although ulnar-sided sclerosis of the lunate may result from UIS, it has also been observed in asymptomatic patients. Rhee et al. [27] compared 33 patients with UIS with 375 asymptomatic patients and reported that lunate subchondral cysts were more frequently identified in patients with UIS (57.6%) than in the normal population (10.4%). They concluded that a positive UV was not a major cause of lunate subchondral cyst formation. Moreover, Cha et al. compared patients with residual positive and negative UV after shortening of the osteotomy of UIS [28]. They reported that only 31.1% of patients with UIS had lunate subchondral cysts. Similarly, in our study, only 2 of the 18 patients with ulnar-sided sclerosis of the lunate were symptomatic. These findings indicate that ulnar-sided sclerosis of the lunate is not a major factor associated with UIS.

The prevalence of lunate subchondral cysts in the normal population is unknown because most patients with carpal bone cysts are asymptomatic [29]. A previous study reported that 10.4% of the normal population had lunate subchondral cysts [27]. Lunate cysts are especially prevalent in elderly patients. Similarly, other studies have suggested that subchondral bone cysts are part of the aging process [30,31]. However, the precise pathophysiology of subchondral bone cysts remains unknown. Two theories have been proposed to explain subchondral bone cyst formation. Jeffe et al. [32] suggested the “fluid breach theory”, in which a cyst initially appears as a synovial cyst and then infiltrates the bone. Eiken et al. [33] proposed the “bone contusion theory”, which states that overloading causes bones to die and liquefy in areas that exceed the limits of physiological endurance. They reported that pain was correlated with cysts with marginal sclerosis. Others proposed that the two theories can coexist, indicating that there are two types of cysts [29]. Although our study did not prove the pathophysiology of subchondral sclerosis or cysts, we agree that two types of cysts exist. In this study, the prevalence of ulnar-sided sclerosis of the lunate, with or without cysts, was 4.88%, lower than the rate reported in a previous study. Because we believe that ulnar-sided subchondral sclerosis with lunate cysts in UIS may have been caused by the bone contusion theory proposed by Eiken et al. [33], we excluded patients who had a subchondral cyst without sclerosis of the lunate from this study.

This study had several limitations. First, it was limited by its retrospective design, and the sample sizes of the experimental and control groups were too small to enable us to draw any significant conclusions. This was partially due to the low incidence of ulnar-sided sclerosis of the lunate: approximately 4.8–10%. However, the authors attempted to overcome this limitation by using a matched case-control study design. Second, in this study, the presence of ulnar-sided sclerosis was confirmed by plain radiography, and CT and radiographic parameters were measured in posteroanterior and lateral views of the distal radius, which could have led to bias among the observers. To overcome this bias, two independent experienced orthopedic surgeons repetitively confirmed ulnar-sided sclerosis of the lunate and performed radiographic measurements at 2-week intervals. Finally, because preoperative ulnar-sided wrist pain was documented based on patient recall, it is unclear whether ulnar-sided wrist pain before trauma was caused by UIS.

## 5. Conclusions

The presence of subchondral sclerosis with or without cystic changes in the proximal ulnar portion of the lunate, as evidenced on plain radiographs prior to VLPF for DRF, did not increase ulnar-sided wrist pain or affect the postoperative clinical outcomes if anatomical reduction and stable fixation of the fracture were achieved, even if the postoperative UV was slightly increased during follow-up after VLPF.

## Figures and Tables

**Table 1:** Comparison of demographic characteristics between the groups.

	Group A ^†^ (*n* = 18)	Group B ^‡^ (*n* = 72)	*p*-Value
Sex (female, %)	13 (72.2%)	48 (66.7%)	0.652
Age (year)	67.06 ± 9.01	62.10 ± 12.88	0.128
Fracture type			0.894
Type A	1 (5.6%)	4 (5.6%)	
Type B	4 (22.2%)	14 (19.4%)	
Type C	13 (72.2%)	54 (75.0%)	
Follow-up period (months)	17.51 ± 6.62	16.32 ± 5.29	0.424

^†^ Group A, patients who had ulnar-sided sclerotic lesions of lunate; ^‡^ Group B, patients who were not sclerotic.

**Table 2:** Comparison of clinical outcomes between the groups.

	Group A ^†^ (*n* = 18)	Group B ^‡^ (*n* = 72)	*p*-Value	Cohen’s d
Ulnar-sided wrist pain (*n*)	2 (11.1%)	3 (4.1%)		
VAS				
PO 6 months	0.7 (0–4)	0.6 (0–3)	0.672	0.102
PO 12 months	0.5 (0–3)	0.4 (0–2)	0.593	0.124
DASH				
PO 6 months	7.2 (0–25.8)	7.1 (0–18.5)	0.795	0.03
PO 12 months	5.6 (0–16.8)	4.2 (0–11.5)	0.882	0.48
Grip strength(% compared to contra.)				
PO 6 months	85.5% (80.5–98.2)	90.9% (82.3–98.5)	0.349	1.46
PO 12 months	88.5% (80.2–100)	92.2% (82.6–100)	0.687	0.9

VAS—visual analogue scale; DASH—disability of the arm, shoulder, and hand; ^†^ Group A, patients who had ulnar-sided sclerotic lesions of lunate; ^‡^ Group B, patients who had no sclerotic lesion of lunate.

**Table 3:** Comparison of postoperative radiographic parameters between the groups.

	Group A ^†^ (*n* = 18)	Group B ^‡^ (*n* = 72)	*p*-Value	Cohen’s d
UV at 2 weeks after surgery	2.0 ± 2.4	0.4 ± 2.3	0.010 *	0.690
UV at last follow-up	2.6 ± 3.0	0.6 ± 2.2	0.007 *	0.842
ΔUV (mm)	0.6 ± 1.2	0.5 ± 1.0	0.225	0.096
RI (°) at last follow-up	21.7 ± 3.1	24.4 ± 3.7	0.055	0.752
RL (mm) at last follow-up	10.3 ± 1.6	11.4 ± 1.7	0.113	0.654
VT (°) at last follow-up	6.6 ± 7.6	8.9 ± 6.5	0.195	0.341

UV—ulnar variance; ΔUV (mm), last UV—postop 2 weeks UV; RI—radial inclination; RL—radial length; VT—volar tilt, ^†^ Group A, patients who had ulnar-sided sclerotic lesions of lunate; ^‡^ Group B, patients who had no sclerotic lesion of lunate. * Statistically significant (*p* < 0.05).

## Data Availability

The authors agree to share their raw data, any digital study materials, and analysis codes as appropriate.

## References

[1] Chung K.C., Spilson S.V. (2001). The frequency and epidemiology of hand and forearm fractures in the United States. J. Hand Surg. Am..

[2] Lee J.H., Lee J.K., Park J.S., Kim D.H., Baek J.H., Kim Y.J., Yoon K.T., Song S.H., Gwak H.G., Ha C. (2020). Complications associated with volar locking plate fixation for distal radius fractures in 1955 cases: A multicentre retrospective study. Int. Orthop..

[3] Kim J.K., Kim D.J., Yun Y. (2016). Natural history and factors associated with ulnar-sided wrist pain in distal radial fractures treated by plate fixation. J. Hand Surg. Eur. Vol..

[4] Sachar K. (2012). Ulnar-sided wrist pain: Evaluation and treatment of triangular fibrocartilage complex tears, ulnocarpal impaction syndrome, and lunotriquetral ligament tears. J. Hand Surg. Am..

[5] Stockton D.J., Pelletier M.E., Pike J.M. (2015). Operative treatment of ulnar impaction syndrome: A systematic review. J. Hand Surg. Eur. Vol..

[6] Sammer D.M., Rizzo M. (2010). Ulnar impaction. Hand Clin..

[7] Imaeda T., Nakamura R., Shionoya K., Makino N. (1996). Ulnar impaction syndrome: MR imaging findings. Radiology.

[8] Terzis A., Koehler S., Sebald J., Sauerbier M. (2020). Ulnar shortening osteotomy as a treatment of symptomatic ulnar impaction syndrome after malunited distal radius fractures. Arch. Orthop. Trauma Surg..

[9] Aibinder W.R., Izadpanah A., Elhassan B.T. (2018). Ulnar Shortening Versus Distal Radius Corrective Osteotomy in the Management of Ulnar Impaction After Distal Radius Malunion. Hand.

[10] Bu J., Patterson R.M., Morris R., Yang J., Viegas S.F. (2006). The effect of radial shortening on wrist joint mechanics in cadaver specimens with inherent differences in ulnar variance. J. Hand Surg. Am..

[11] Esenwein P., Sonderegger J., Gruenert J., Ellenrieder B., Tawfik J., Jakubietz M. (2013). Complications following palmar plate fixation of distal radius fractures: A review of 665 cases. Arch. Orthop. Trauma Surg..

[12] Ma H.H., Chen Y.C., Huang H.K., Huang Y.C., Chang M.C., Wang J.P. (2022). Comparing radial lengthening osteotomy with ulnar shortening osteotomy to treat ulnar impaction syndrome after distal radius fracture malunion. Arch. Orthop. Trauma Surg..

[13] Stirling P.H.C., Oliver W.M., Ng N., Oliver C.W., McQueen M.M., Molyneux S.G., Duckworth A.D. (2023). Distal radius malunion: Outcomes following an ulnar shortening osteotomy. Eur. J. Orthop. Surg. Traumatol..

[14] Arora R., Lutz M., Hennerbichler A., Krappinger D., Espen D., Gabl M. (2007). Complications following internal fixation of unstable distal radius fracture with a palmar locking-plate. J. Orthop. Trauma.

[15] Arora R., Lutz M., Fritz D., Zimmermann R., Oberladstatter J., Gabl M. (2005). Palmar locking plate for treatment of unstable dorsal dislocated distal radius fractures. Arch. Orthop. Trauma Surg..

[16] Cheng M.F., Chiang C.C., Lin C.C., Chang M.C., Wang C.S. (2021). Loss of radial height in extra-articular distal radial fracture following volar locking plate fixation. Orthop. Traumatol. Surg. Res..

[17] Quadlbauer S., Pezzei C., Jurkowitsch J., Rosenauer R., Pichler A., Schättin S., Hausner T., Leixnering M. (2020). Functional and radiological outcome of distal radius fractures stabilized by volar-locking plate with a minimum follow-up of 1 year. Arch. Orthop. Trauma Surg..

[18] (2018). Radius and Ulna. J. Orthop. Trauma.

[19] Medoff R.J. (2005). Essential radiographic evaluation for distal radius fractures. Hand Clin..

[20] Sachar K. (2008). Ulnar-sided wrist pain: Evaluation and treatment of triangular fibrocartilage complex tears, ulnocarpal impaction syndrome, and lunotriquetral ligament tears. J. Hand Surg. Am..

[21] Shin A.Y., Deitch M.A., Sachar K., Boyer M.I. (2005). Ulnar-sided wrist pain: Diagnosis and treatment. Instr. Course Lect..

[22] Baek G.H., Chung M.S., Lee Y.H., Gong H.S., Lee S., Kim H.H. (2006). Ulnar shortening osteotomy in idiopathic ulnar impaction syndrome. Surgical technique. J. Bone Jt. Surg. Am. Vol..

[23] Palmer A.K., Werner F.W. (1984). Biomechanics of the distal radioulnar joint. Clin. Orthop. Relat. Res..

[24] Werner F.W., Palmer A.K., Fortino M.D., Short W.H. (1992). Force transmission through the distal ulna: Effect of ulnar variance, lunate fossa angulation, and radial and palmar tilt of the distal radius. J. Hand Surg. Am..

[25] Tomaino M.M. (1998). Ulnar impaction syndrome in the ulnar negative and neutral wrist. Diagnosis and pathoanatomy. J. Hand Surg. Br..

[26] Tatebe M., Nakamura R., Horii E., Nakao E. (2005). Results of ulnar shortening osteotomy for ulnocarpal impaction syndrome in wrists with neutral or negative ulnar variance. J. Hand Surg. Br..

[27] Rhee S.M., Lee J.Y., Song K.S., Lee G.Y., Lee J.S. (2019). Lunate subchondral cysts: Are there specific radiologic findings for patients with symptomatic ulnocarpal impaction?. J. Orthop. Sci..

[28] Cha S.M., Shin H.D., Kim K.C. (2012). Positive or negative ulnar variance after ulnar shortening for ulnar impaction syndrome: A retrospective study. Clin. Orthop. Surg..

[29] Ikeda M., Oka Y. (2000). Cystic lesion in carpal bone. Hand Surg..

[30] Li G., Yin J., Gao J., Cheng T.S., Pavlos N.J., Zhang C., Zheng M.H. (2013). Subchondral bone in osteoarthritis: Insight into risk factors and microstructural changes. Arthritis Res. Ther..

[31] Ding M., Odgaard A., Linde F., Hvid I. (2002). Age-related variations in the microstructure of human tibial cancellous bone. J. Orthop. Res..

[32] Jaffe H.L., Selin G. (1951). Tumors of bones and joints. Bull. N. Y Acad. Med..

[33] Eiken O., Jonsson K. (1980). Carpal bone cysts: A clinical and radiographic study. Scand. J. Plast. Reconstr. Surg..

